# Multi-vehicle detection with identity awareness using cascade Adaboost and Adaptive Kalman filter for driver assistant system

**DOI:** 10.1371/journal.pone.0173424

**Published:** 2017-03-15

**Authors:** Baofeng Wang, Zhiquan Qi, Sizhong Chen, Zhaodu Liu, Guocheng Ma

**Affiliations:** Laboratory of Vehicle Engineering, School of Mechanical Engineering, Beijing Institute of Technology, Beijing 100081, China; Chongqing University, CHINA

## Abstract

Vision-based vehicle detection is an important issue for advanced driver assistance systems. In this paper, we presented an improved multi-vehicle detection and tracking method using cascade Adaboost and Adaptive Kalman filter(AKF) with target identity awareness. A cascade Adaboost classifier using Haar-like features was built for vehicle detection, followed by a more comprehensive verification process which could refine the vehicle hypothesis in terms of both location and dimension. In vehicle tracking, each vehicle was tracked with independent identity by an Adaptive Kalman filter in collaboration with a data association approach. The AKF adaptively adjusted the measurement and process noise covariance through on-line stochastic modelling to compensate the dynamics changes. The data association correctly assigned different detections with tracks using global nearest neighbour(GNN) algorithm while considering the local validation. During tracking, a temporal context based track management was proposed to decide whether to initiate, maintain or terminate the tracks of different objects, thus suppressing the sparse false alarms and compensating the temporary detection failures. Finally, the proposed method was tested on various challenging real roads, and the experimental results showed that the vehicle detection performance was greatly improved with higher accuracy and robustness.

## Introduction

Advanced driver assistance systems for cars like forward collision warming(FCW), autonomous emergency braking(AEB) and adaptive cruise control(ACC), require a reliable perception of the preceding vehicles. Different sensors including radar, Lidar, stereo vision and monocular vision have been researched for vehicle detection. With the availability of low-cost-high-performance cameras and feasible computer vision technologies, vision based vehicle detection methods are becoming an active area of research in recent years [1]. In this paper, we focus on the research of monocular-based multi-vehicle detection.

Robust vehicle detection using monocular vision is a challenging problem. Traditional methods tended to use priori knowledge of vehicle rears such as edge [2], shadow [3], and symmetry [4] to hypothesized the location of the vehicles in the image plane. These methods has been verified to be effective and fast in simple situations, but cannot achieve a satisfying detection under complex scenarios such as pose change, illumination variation, noisy background, etc. Motion cues such as optical flow have also been used for vehicle detection [5]. But these methods require a relatively stable background, and are more suitable for traffic surveillance with static cameras or stereo-vision based method. With the development of machine learning and data mining technology, the machine learning based methods are becoming more and more popular in different research field [6–8]. For vision based vehicle detection, modern methods often employ different classification models including Support Vector Machine(SVM), Adaboost, deep nerual networks etc., with higher level local features, such as Histogram of Oriented Gradient(HOG), Haar-like features and Convolutional Neural Networks features, thus to locate the vehicles more robustly under various challenging conditions.

However, like all the other detectors, the measurements given by vision-based algorithm also come with occasional detection failures and false alarms due to various environments. To improve the detection performance, different approaches have been proposed. Trivedi et al. [10] developed a vehicle detector based on Adaboost classification and Haar-like features, and proposed an active-learning framework to improve the discriminating capacity of the classifier through iterative training with the ambiguous misclassified samples. Stanciulescu et al. [11] improved the classifier through introducing new Adaboost features that extended by the topological properties of vehicle rear appearance. Kim [12] proposed Haar-like edge features to work in cooperation with the traditional Haar-like intensity features, and efficiently reduced the false detections. Context of the driving scene is another efficient way to improve the detection accuracy, and among which the vanishing point(VP) is the most popular cue to filter out the vehicle hypotheses with locations above it [13]. But the limitation of this approach is that the false alarms below VP cannot be filtered out.

The aforementioned methods in general focus mainly on improving detection performance on a single frame, while further improvement could be achieved by a tracking mechanism through filtering from multiple frames. Vehicle tracking has been addressed by many researchers, different methods such as Kalman filter(KF), extended Kalman filter(EKF) and particle filter(PF) have been applied, and KF is especially popular due to its simplicity and effectiveness [4, 12]. Generally, KF were used to estimate the possible region of interest(ROI) for vehicle redetection [4], compensate the temporary detection losses, and smooth the jittery trajectory [12]. However in real world vehicle tracking scenario, there are two limitations of conventional KF. First, conventional KF requires a precise prior knowledge of the the process noise and measurement noise properties(i.e. the matrices *Q* and *R*, respectively) which are usually pre-set and remain constant throughout the whole tracking process [14, 15]. Because of changing distance and scale when approaching or departing the front vehicles, the noise properties of vehicle objects vary greatly, which will seriously degrade the filtering performance and may even result in filter divergence during long-term tracking [16]. Secondly, aforementioned approaches mainly focused on how to predict and update the state of the target vehicles with measurements, but didn’t discuss how to resolve the identity assigning problem of different objects across frames. In fact, scenarios with multi-vehicle are quite common in real road driving, and the identity awareness between observations and measurements is the foremost issue for a multi-object filtering mechanism. Additionally, the changing number of vehicle targets in the field of view(FOV) as well as the occasional detection misses and sparse false alarms make the association procedure more challenging.

**Fig 1:**
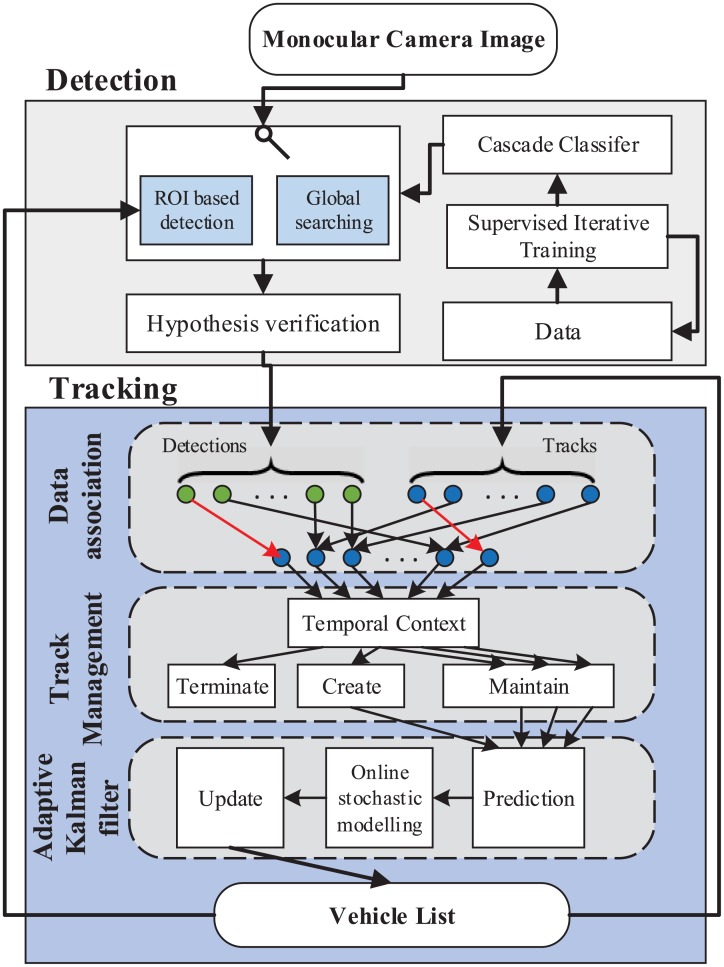
Flow chart of multi-vehicle detection and tracking.

Based on the points discussed above, we presented an improved multi-vehicle detection and tracking method using cascade Adaboost and adaptive Kalman filter(AKF) with target identity awareness in this paper. The outline of the proposed method is shown in Fig 1. Vehicles are detected by a cascade Adaboost classifier using Haar-like features, followed by a more comprehensive verification process which could refine the vehicle hypothesis in terms of both location and dimension. In vehicle tracking, each vehicle is tracked with identity awareness by AKF in collaboration with a data association approach. The AKF adapts the system noise property through on-line stochastic modelling, the data association correctly assigns different measurements with observations using global nearest neighbour(GNN) algorithm while considering the local validation. During tracking, a temporal context based track management is proposed to decide whether to initiate, maintain or terminate the tracks of different objects. Finally, the proposed method was tested on various challenging real roads, and the experimental results showed that the vehicle detection performance was greatly improved.

The remainder of this paper is organized as follows: the section “Cascade Adaboost for vehicle detection” details the vehicle hypothesis generation and verification method. In the section “Multi-vehicle tracking with identity awareness”, the adaptive Kalman filter with on-line stochastic modelling, the data association method for identity assignment, and the track management method are presented. The experiment results and system performance evaluation of different scenarios are presented in “Experiments”. Conclusions and future works are discussed in “Conclusion”.

## Cascade Adaboost for vehicle detection

### Cascade classifier based on Haar-like features

**Fig 2:**
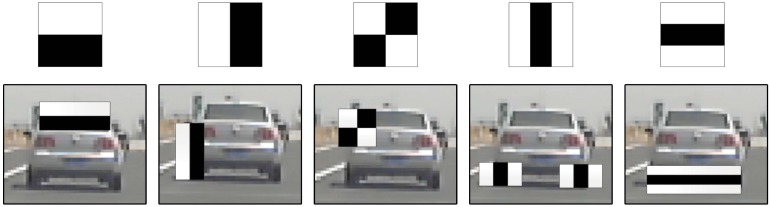
Haar-like features. Top row: basic forms of Haar-like features. Bottom row: vehicle rear appearances suitable for Haar-like features.

Haar-like features based boosted cascade was first introduced by Viola and Jones for face detection [9]. As shown in Fig 2, Haar-like features are a set of different combinations of adjacent rectangles with various scales, positions and aspect ratios, whose value is the difference of sum of intensities between the black area and white area, given by

$$f=\sum_{\left(m,n\right)\in R_{w}}I(m,n)-\sum_{\left(m,n\right)\in R_{b}}I(m,n)$$ (1)

where *R*_*w*_ and *R*_*b*_ represent white area and black area respectively. This value indicates certain characteristics of the image area such as edge, textures and symmetry, thus could be used to encode the local appearance of objects. Given the fact that the appearance of the vehicle rear typically maintains some consistent edges, rectangles, shadows and symmetries as shown in Fig 2, Haar-like features is well-suited for vehicle appearance description.

Let *F* ∝ {*f*_1_, ⋯, *f*_*Nf*_} represent the Haar-like feature family, and S the training set with *N*_*s*_ training samples. In the training process, each image sample of the training set is represented by Haar-like feature family $S\propto \{\left(F_{i},L_{i}\right)|i\in \left\{1,2,\cdots Ns\right\},\;L_{i}\in \pm 1\}$, where *L*_*i*_ is the sample label with 1-vehicle and -1-non-vehicle. The extracted features are selected as weak filters by Adaboost through linear combination and weighting to generate a stronger classifier which minimizing the classification error,

$${\hat{c}}_{w}=\arg\min\sum_{i=1}^{N_{s}}c_{w}\left(F_{i}\right)$$ (2)

**Fig 3:**
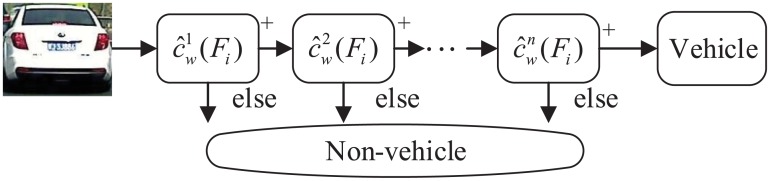
Cascade Adaboost classifier for vehicle detection.

Given an image patch with Haar features of *F*_*i*_, if ${\hat{c}}_{w}\left(F_{i}\right)>0$ it will be classified as vehicle by this classifier. Through iterative training, a cascade of Adaboost classifiers are generated and structured stage by stage into a binary decision tree as shown in Fig 3. The image patches surviving the last classifier stage will be taken as vehicle hypotheses. For efficiency, the less accurate but faster classifiers are arranged in earlier stages, so as to reject the obvious non-vehicles with little processing. The confidence of a vehicle hypothesis could be defined as

$$C_{w}\left(F_{i}\right)=\sum_{j=1}^{n}{\hat{c}}_{w}^{j}\left(F_{i}\right)$$ (3)

where *n* is the number of the stages of the cascade classifier.

### Dataset collection and classifier training

**Fig 4:**
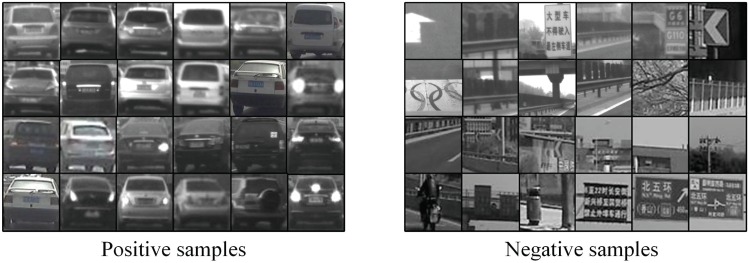
Training samples for classifier.

Training samples are generally collected by manual cropping, which is a very labour-cost procedure. In data collection for classifier training, we adopted a coarse to fine rule. First, a certain amount of samples were manually cropped to hatch a initial classifier. Afterwards, the classifier was run on new data to generate more samples for retraining. Compared with manually cropping the raw image, the detection results are more easily to select and process, thus a massive samples could be collected with less time and labour cost. The collected sample were used for retraining to generate a more advanced classifier. Through fast iterative training on booming data under the supervision of people, the performance of the classifier could be improved rapidly. In this case, we carefully cropped 2881 positive samples and 6650 negative samples for initial training and normalized them into 20 × 20 form. After 4 rounds of iterative training, a cascade vehicle detector with 20 stages was generated after training on 23641 positive samples and 41562 negative samples in total. Some of the training samples are as shown in Fig 4.

### Hypothesis generation

**Fig 5:**
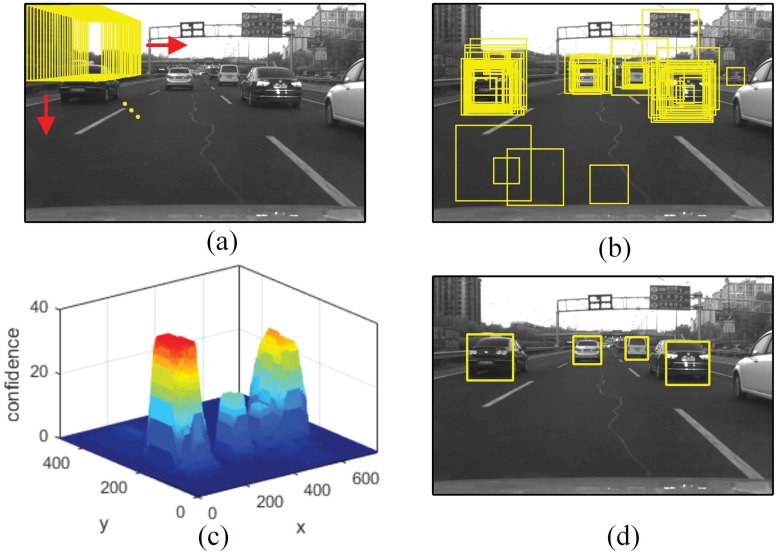
Vehicle detection scheme. (a)Sliding window sampling. (b)Raw detection results. (c)Confidence map of raw detections. (d)Final results after redundant boxes fusion.

In vehicle hypothesis generation, cascade classifier adopts a sliding window approach. As shown in Fig 5(a), the window scans the image globally at all scales and locations to sample patches which are then checked by the classifier cascade. Since the classifier tolerates a wider region misalignment, several redundant windows near the ideal location of a vehicle object will be returned with different confidences, as shown in Fig 5(b) and 5(c). The raw detection results are then grouped by clustering and non-maxima suppression method [17], and finally vehicle hypotheses are generated in the form of squared bounding boxes as shown in Fig 5(d), which could be given by,

$$v_{i}\;=\;\left[\begin{array}{ccc} x_{i} & y_{i} & w_{i} \end{array}\right],\;i\;=\;1,2,3,\cdots \;$$ (4)

where *i* is the index of the object in the list, (*x*_*i*_, *y*_*i*_) is defined as the center of bottom line of the bounding box, and *w*_*i*_ is the side length of the bounding box.

The global sliding window approach is too time-consuming for real-time application. To improve the processing speed, we adopted the ROI based method to narrow down the search region according to the detection results of last frame. To detect the new appearing targets in time, the global searching is applied periodically every several frames. The period of global searching is experimentally set as 5 frames in this paper. Considering the average processing speed of our method(discussed in the Experiments section), the driving scene will be globally scanned about every 230ms, which is very reliable for the ADAS system to find new appearing vehicles in time.

### Hypothesis refinement

Raw vehicle hypotheses are usually mixed with some false detections, therefore a post-processing is very essential to verify the hypotheses. It is a priori knowledge that that the widths of different vehicles(from cars to truck) are within in a certain range(about 1.5m–2.5m), and all the vehicles are located on the road. Therefore the the raw hypotheses that violate this constraint could be safely filtered out as false detections. To get the real width of a vehicle hypothesis, the projection model of camera is used,

$$\left[\begin{array}{c} x_{I}\\ y_{I}\\ 1 \end{array}\right]=\underset{K}{\underset{︸}{\left[\begin{array}{ccc} f_{cu} & \alpha_{c}f_{cu} & c_{u}\\ 0 & f_{cv} & c_{v}\\ 0 & 0 & 1 \end{array}\right]}}\underset{R}{\underset{︸}{\left[\begin{array}{ccc} r_{11} & r_{12} & r_{13}\\ r_{21} & r_{22} & r_{23}\\ r_{31} & r_{32} & r_{33} \end{array}\right.}}\underset{T}{\underset{︸}{\left.\begin{array}{c} t_{1}\\ t_{2}\\ t_{3} \end{array}\right]}}\;\left[\begin{array}{c} x_{w}\\ y_{w}\\ z_{w}\\ 1 \end{array}\right]$$ (5)

where [*x*_*I*_, *y*_*I*_]^*T*^ is the image coordinates, [*x*_*w*_, *y*_*w*_, *z*_*w*_]^*T*^ is the corresponding real world location *K* is the intrinsic matrix with (*f*_*cu*_, *f*_*cu*_) the focal lengths expressed in pixels units vertically and horizontally, (*c*_*u*_, *c*_*v*_) the principal point, and *α*_*c*_ the skew coefficient. *R* and *T* are the extrinsic parameters defining the rotation and translation from world coordinate to camera coordinate. These parameters could be calibrated once the camera is installed in the vehicle. Theoretically, the projection transformation is irreversible due to the loss of depth information, however based on the flat road assumption [18], Eq 5 could be simplified as

$$\left[\begin{array}{c} x_{I}\\ y_{I}\\ 1 \end{array}\right]=\underset{M}{\underset{︸}{\left[\begin{array}{ccc} f_{cu} & \alpha_{c}f_{cu} & c_{u}\\ 0 & f_{cv} & c_{v}\\ 0 & 0 & 1 \end{array}\right]\;\left[\begin{array}{ccc} r_{11} & r_{12} & t_{1}\\ r_{21} & r_{22} & t_{2}\\ r_{31} & r_{32} & t_{3} \end{array}\right]}}\left[\begin{array}{c} x_{w}\\ y_{w}\\ 1 \end{array}\right]$$ (6)

This simplified projection model is reversible, and given a vehicle hypothesis *v*_*i*_ = [*x*_*i*_, *y*_*i*_, *w*_*i*_], its width in real world could be estimated,

$${\bar{w}}_{w}\;=\;\left|M^{-1}\;\left[x_{i}-\frac{w_{i}}{2},y_{i},1\right]-M^{-1}\;\left[x_{i}+\frac{w_{i}}{2},y_{i},1\right]\right|$$ (7)

**Fig 6:**
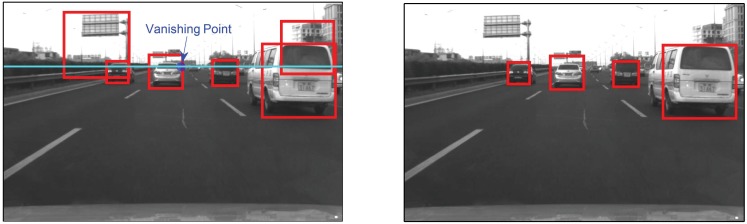
Hypothesis refinement result. (Left) Vanishing point based method fails to filter the false hypotheses with bottom edge under vanish point; (Right) Proposed method successfully filters out the false hypothesis.

Eq 7 shows that the estimated width ${\bar{w}}_{w}$ takes into consideration of both the location and dimension of the vehicle hypothesis. According to the prior knowledge, only the vehicle hypotheses with proper location and dimension will have a estimated width within the reasonable range, while the others violating this constraint could be safely eliminated as false alarms. In this paper, we define the with range as [*w*^−^, *w*^+^], where *w*^−^ and *w*^+^ are the predefined upper and the lower width threshold of the vehicle. To account for the measurement uncertainty, we relax the width range of vehicle as [1.2, 3]*m* through experiments. Compared with traditionally used vanishing point for vehicle hypothesis refinement, this constraint could provide a more comprehensive verification and filter out more false detections, see Fig 6 for example.

## Multi-vehicle tracking with identity awareness

In this section, the raw measurements detected by the vehicle detector are filtered by a multi-object tracking mechanism. In this tracking mechanism, the AKF with on-line stochastic modelling is developed for the state filtering of each object, a GNN based approach with local validation is used for data association, and a temporal context based track management is built to create, maintain, or terminate the tracking each object based on the temporal context of their detection histories.

### Adaptive Kalman filter

With the detection result given by the detector, we define the sate vector of a vehicle object *v*_*i*_(*k*) at the discrete time instant *k* as

$$X_{i}\left(k\right)=\left[x_{i}\left(k\right),\;y_{i}\left(k\right),\;w_{i}\left(k\right),\;{\dot{x}}_{i}\left(k\right),\;{\dot{y}}_{i}\left(k\right),\;{\dot{w}}_{i}\left(k\right)\right]$$ (8)

where the definitions of the parameters are the same as in Eq 4. Since the time intervals between two successive frames are very small due to the high acquisition frequency of camera, the inter-frame state change of moving targets could be considered as uniform motion. Thus the system and measurement models of the vehicle are defined as follows:

$$X_{i}\left(k\right)=AX_{i}\;\left(k-1\right)+\omega_{i}\;\left(k-1\right),\;\omega_{i}\;\left(k-1\right)\propto N\;\left(0,Q_{k-1}\right)$$ (9)

$$Z_{i}\;\left(k\right)=CX_{i}\;\left(k\right)+\upsilon_{i}\;\left(k\right),\;\upsilon_{i}\;\left(k\right)\propto N\;\left(0,R_{k}\right)$$ (10)

where *ω*_*i*_(*k*) is the white Gaussian process noise with covariance of *Q*(*k* − 1), *υ*_*i*_(*k*) is the white Gaussian measurement noise with the covariance of *R*(*k*), *A* and *C* are the state transition matrix and measurement matrix, which could be defined respectively as,

$$A=\left[\begin{array}{cccccc} 1 & 0 & 0 & \Delta t & 0 & 0\\ 0 & 1 & 0 & 0 & \Delta t & 0\\ 0 & 0 & 1 & 0 & 0 & \Delta t\\ 0 & 0 & 0 & 1 & 0 & 0\\ 0 & 0 & 0 & 0 & 1 & 0\\ 0 & 0 & 0 & 0 & 0 & 1 \end{array}\right]$$ (11)

$$C=\left[\begin{array}{cccccc} 1 & 0 & 0 & 0 & 0 & 0\\ 0 & 1 & 0 & 0 & 0 & 0\\ 0 & 0 & 1 & 0 & 0 & 0 \end{array}\right]$$ (12)

where Δ*t* is the time of the frame interval.

As mentioned above, the KF pre-sets the process noise and measurement noise, neglecting the noise level change which is very common in vision based vehicle tracking due to changing distance and scale. When the stochastic properties of the process and measurement noises becomes improper, large bias or oscillation will occur. To remedy the effect caused by changing noise properties, we built an AKF filter which could adapt the properties of measure and system noise covariance in on-line manners. The proposed filter is composed of three steps: prediction, on-line stochastic modelling and update.

#### Prediction step

The priori state and the state covariance of the vehicle in the following frame is predicted,

$$X_{i}\left(k|k-1\right)=AX_{i}\left(k-1|k-1\right)$$ (13)

$$P_{i}\left(k|k-1\right)=AP_{i}\left(k-1|k-1\right)A^{T}+Q_{i}\left(k-1\right)$$ (14)

where *X*_*i*_(*k*|*k* − 1) and *P*_*i*_(*k*|*k* − 1) denote the predicted state vector and innovation covariance of next epoch respectively, *X*_*i*_(*k* − 1|*k* − 1) and *P*_*i*_(*k* − 1|*k* − 1) are the filtered state vector of last epoch.

#### On-line stochastic modelling

Before updating the system directly, we modelled the properties of the measurement noise based on the innovation sequence which could be defined as

$$\begin{array}{ll} \varepsilon_{i}\;\left(k\right) & =Z_{i}\;\left(k\right)-CX_{i}\;\left(k|k-1\right)\;\;=\;\;CX_{i}\;\left(k\right)+\upsilon_{i}\;(k)-CX_{i}\;\left(k|k-1\right)\\ & =\;\;C\left[X_{i}\;\left(k\right)-X_{i}\;\left(k|k-1\right)\right]+\upsilon_{i}\;\left(k\right) \end{array}$$ (15)

where *Z*_*i*_(*k*) is the real measurement. It could be seen that the innovation reflects the difference between the prediction and the real measurements, and is a zero-mean Gaussian with noise sequence. Taking variances on both sides, we could obtain the innovation covariance as,

$$\begin{array}{cc} C_{\varepsilon_{i}}\left(k\right)= & E\;\left[\varepsilon_{i}\;\left(k\right)\;\varepsilon_{i}{\left(k\right)}^{T}\right]=CP_{i}\;\left(k|k-1\right)C^{T}+R_{k}\\ = & C\;\left[AP_{i}\;\left(k-1|k-1\right)A^{T}+Q_{i}\;\left(k-1\right)\right]C^{T}+R_{k} \end{array}$$ (16)

To get some statistical smoothing, the calculation of covariance is carried out through averaging the innovation samples inside a moving window at epoch *k*,

$$C_{\varepsilon_{i}}\left(k\right)\;=\;\frac{1}{W_{m}}\sum_{i=0}^{W_{m}-1}\varepsilon_{i}\left(k-i\right)\;\varepsilon_{i}{\left(k-i\right)}^{T}$$ (17)

where *W*_*m*_ is the size of the window. From Eq 12 to Eq 14, the measurement covariance could be adaptively estimated as

$$R_{i}\left(k\right)=C_{\varepsilon_{i}}\left(k\right)-CP_{i}\;\left(k|k-1\right)C^{T}$$ (18)

#### Update step

With the estimated measurement covariance, a posteriori state is estimated based on Bayes’ law in the update step as follows,

$$K_{i}\left(k\right)=P_{i}\left(k|k-1\right)C^{T}{\left[CP_{i}\left(k|k-1\right)C^{T}+R_{i}\left(k\right)\right]}^{-1}$$ (19)

$$X_{i}\left(k|k\right)=X_{i}\;\left(k|k-1\right)+K_{i}\;\left(k\right)\;\left[Z_{i}\;\left(k\right)-CX_{i}\;\left(k|k-1\right)\right]$$ (20)

$$P_{i}\left(k|k\right)=\left[I-K_{i}\;\left(k\right)\;C\right]\;P_{i}\;\left(k|k-1\right)$$ (21)

where *K*_*i*_(*k*) is the Kalman gain, which defines the updating weight between the new measurement and the prediction from the system dynamic model. Based on the residual between the measurement and filtered states, the process noise *Q*_*i*_ (*k*) is also estimated for updating,

$$Q_{i}\;\left(k\right)=\frac{1}{m}\sum_{i=0}^{m-1}\Delta X\;\left(k-i\right)\Delta X{\left(k-i\right)}^{T}+P_{i}\;\left(k|k\right)-AP_{i}\;\left(k-1|k-1\right)A^{T}$$ (22)

where Δ*X* (*k* − *i*) = *X*_*i*_ (*k*) − *X*_*i*_ (*k*|*k* − 1). With the on-line stochastic modelling, the properties of the process noise and the measurement noise used in the filter are adaptively updated so as to avoid the filtering divergence caused by changing dynamics during vehicle tracking.

### Data association

In multiple objects tracking, the problem before filtering is to decide the correct identity association between the observations and measurements. Different methods such as nearest-neighbouring(NN) [19], jointed probability data association(JPDAF) [20], and multiple hypotheses tracker(MHT) [21] have been proposed for multi-target association. Considering the efficiency and reliability, we adopted the global nearest neighbour(GNN) [22] approach for data association while considering the local validity.

The data association problem could be formulated as a linear assignment problem which searches one-to-one mapping solution between the detection list of *m* measurements and the track list of *n* tracked objects,

$$\begin{array}{c} \hat{H}\left(k\right)=\arg\max\sum_{j=1}^{m}\sum_{i=1}^{n}l_{k}\left(i,j\right)•h_{k}\left(i,j\right)\\ H\left(k\right)=\left[\begin{array}{cccc} h_{k}\left(1,1\right) & h_{k}\left(1,2\right) & \cdots & h_{k}\left(1,m\right)\\ h_{k}\left(2,1\right) & h_{k}\left(2,2\right) & \cdots & h_{k}\left(2,m\right)\\ \vdots & \vdots & \cdots & \vdots \\ h_{k}\left(n,1\right) & h_{k}\left(n,2\right) & \cdots & h_{k}\left(n,m\right) \end{array}\right]\\ s.t.\left\{\begin{array}{c} \sum_{i=1}^{n}h_{k}\left(i,j\right)\in \left\{0,1\right\},\\ \sum_{j=1}^{m}h_{k}\left(i,j\right)\in \left\{0,1\right\},\\ h_{k}\left(i,j\right)\in \left\{0,1\right\},\\ h_{k}\left(i,j\right)=0,j\in \left\{0,1,\cdots ,m\right\},\text{if}\sum_{i=1}^{n}l_{k}\left(i,j\right)=0,\\ h_{k}\left(i,j\right)=0,i\in \left\{0,1,\cdots ,n\right\},\text{if}\sum_{j=1}^{m}l_{k}\left(i,j\right)=0, \end{array}\right. \end{array}$$ (23)

where *H* is the mapping matrix.

**Fig 7:**
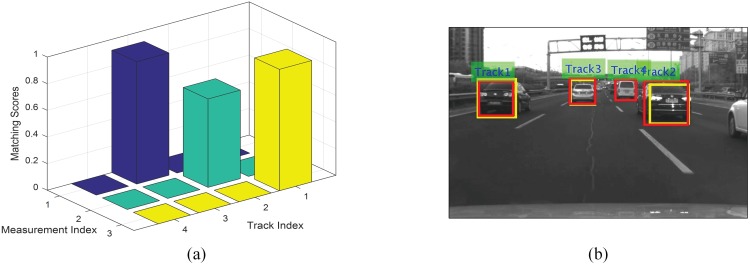
Data association result. (Left) The visualization of the matching confidence matrix. (Right) Association results between the measurements (yellow) and existing tracks (red).

The selection of likeliood should try to maximize the matching degree of correct association hypotheses, while suppressing those of the false ones. Traditional methods usually use the distances(Mahalanobis or Euclidean) of the observation states for evaluation [23]. However this method needs a set of scalars to balance the weights between different state variables(location and dimension), which is not easy to tune. Some more advanced method also used high-dimensional appearance features through on-line learning to handle more challenging scenarios such as hundreds of crowded objects tracking with high occlusions [24]. But these methods seem too luxurious in our case, since preceding vehicles in ADAS scenarios are generally box-shaped, and the vehicles involved in occlusion scenario usually have significant scale differences in 2D image plane(due to their different longitudinal distances) which is very reliable for identity discrimination in cooperation with location. Overlap ratio can measure the similarity of two objects in terms of both the location and dimension without weighting, thus is selected for the matching confidence evaluation in this paper. It is worth to mention that the GNN method does not account for the local validity of one particular association hypothesis, and wrong association result may be generated when partial occlusion between different vehicles exists. To avoid this problem, an independent threshold is imposed to check the local validity of each association hypothesis, therefore the likelihood is defined as follows:

$$l_{k}\;\left(i,j\right)=\left\{\begin{array}{cc} \frac{{\tilde{v}}_{j}\left(k\right)\cap {\hat{v}}_{i}\left(k\right)}{{\tilde{v}}_{j}\left(k\right)\cup {\hat{v}}_{i}\left(k\right)}, & \text{if}\;\frac{{\tilde{v}}_{j}\left(k\right)\cap {\hat{v}}_{i}\left(k\right)}{{\tilde{v}}_{j}\left(k\right)\cup {\hat{v}}_{i}\left(k\right)}\;>\;l_{t}\\ 0, & \text{else}. \end{array}\right.$$ (24)

where *l*_*t*_ is the threshold for local validation. A typical association example is as shown in Fig 7, there are four existing tracks while only three measurements are returned, with the proposed method, the measurements are corrected assigned with corresponding tracks for update.

### Temporal context based track management

Multi-vehicle tracking in real road is a dynamical scene where vehicles are disappearing, appearing in the field of view, and the sparse false detections and detection failures bring it more uncertainty. To track the vehicles more reliably, we have developed a track management approach to decide whether to initiate, maintain or terminate tracks according to the temporal context of their tracking histories.

**Fig 8:**
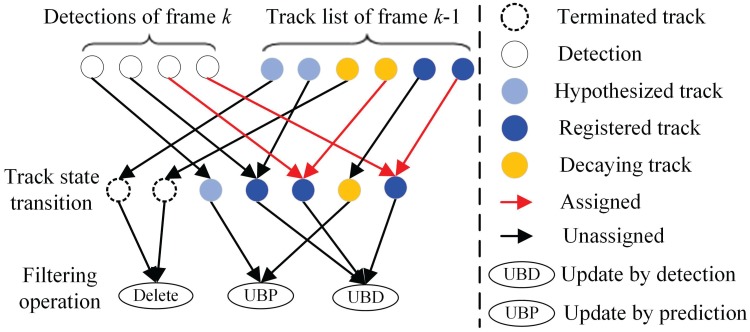
The generic track state transitions in the track management.

In our management system, the detection history of each vehicle is observed simultaneously in the track level,

$$T_{i}\left(k\right)=\left[T_{i}^{C_{+}}\;\left(k\right),\;T_{i}^{C_{-}}\;\left(k\right),\;T_{i}^{S}\;\left(k\right)\right]$$ (25)

where $T_{i}^{C_{+}}\;\left(k\right)$ is the count of consecutive frames with successful detections, $T_{i}^{C_{-}}\;\left(k\right)$ is the count of consecutive frames with detection failures, and $T_{i}^{S}\;\left(k\right)$ is the tack state of the target. In this paper, we defined four track states: $T_{i}^{S}\;\left(k\right)\in \left\{0,1,2,3\right\}$, where $T_{i}^{S}\;\left(k\right)=0$ means terminated track, $T_{i}^{S}\;\left(k\right)=1$ hypothesized track, $T_{i}^{S}\;\left(k\right)=2$ registered track, and $T_{i}^{S}\;\left(k\right)=3$ decaying track. The state transition of each object is driven by the temporal context of their tracking history, which is given by,

$$T_{i}^{S}\left(k\right)=\left\{\begin{array}{cc} 0, & C_{-}^{T}⩽T_{i}^{C_{-}}\;\left(k\right)\\ 1, & 0<T_{i}^{C_{+}}\;\left(k\right)<C_{+}^{T}\\ 2, & C_{+}^{T}⩽T_{i}^{C_{+}}\;\left(k\right)\\ 3, & 0<T_{i}^{C_{-}}\;\left(k\right)<C_{-}^{T} \end{array}\right.$$ (26)

where $C_{+}^{T}$ is the threshold of consecutive successful count which is generally responsible for the track creation and maintenance, and $C_{-}^{T}$ is the the threshold of consecutive failure count which is responsible for track termination. The selection of these thresholds depend on the detection performance(detection rate and false positive rate) of the vehicle detector. An advanced detector could afford more aggressive performance with lower thresholds. While the detector with lower performance may need higher thresholds to suppress the false alarms and compensate the frequent detection failures. In return, the selection of the thresholds also affects the the tracking performance. A higher $C_{+}^{T}$ leads to a lower false alarm rate but a longer delay in the creation of a new track, and a higher $C_{-}^{T}$ will produce a more continuous trajectory but a delayed track termination. The generic state transitions and following filtering operation are as shown in Fig 8. With the proposed track management, the false detections which usually occurs temporarily could be filtered out, and the wrong termination of a tracked vehicle due to momentary unsuccessful detection in one or several frames can also be avoided.

## Experiments

The proposed method was realized by C++/OpenCV and run on a Laptop with Pentium i5–2.5GHz, 2G RAM configuration. A large set of videos with form of 752 × 480 × 8 have been captured under various real road scenarios in Beijing for the test. The performance of vehicle detection is first analysed. Then the tracking performance improvement introduced by AKF is presented through comparison with KF. Finally the multi-vehicle tracking performance in dynamical environment is evaluated.

### Vehicle detection results

**Fig 9:**
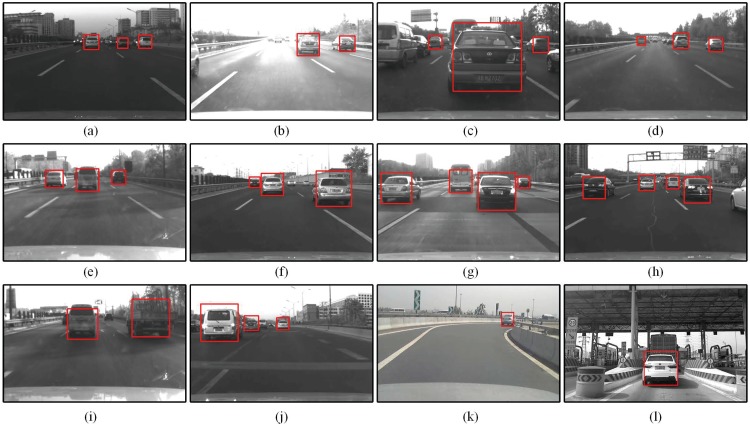
Detection results in different conditions. (a) Dark illumination. (b) Bright illumination. (c) Large scale due to ultra-near distance. (d) Small scale due to long distance. (e) Mottled shadow. (f-g) Slight partial occlusion. (h) Dense traffic. (i)Truck. (j) Van. (k) Strong pose change. (l)Clutter background.

To verify the detection performance of the proposed method, 4 sets of images were chosen as test material. These dataset covers various types of vehicles including sedan, truck, coach, van, and SUV under different scenarios such illumination variation(dark and bright), scale change(large and small), and heavy traffic. Some vehicles with strong pose change and partial occlusion are also covered. Typical detection results of different scenarios are as shown in Fig 9. Table 1 shows the quantitative results of different datasets. The detection results were analysed in terms of true positive rate(TPR), false positive rate(FPR), and false negative rate(FNR). To show the improvement of the proposed hypotheses verification method, the vehicle detector using vanishing point(VDVP) [13] for hypothesis verification is applied for comparison. It is worth to note that vehicles that were too small(smaller than 30 × 30 pixel) and highly occluded(more than half of the rear appearance) were ignored. It could be seen that compared with VDVP the proposed method could achieve a higher TPR(95.0%) with about the same FNR(3.5%), which means the proposed hypothesis verification method is more efficient to eliminate false alarms. Another thing needs to point is that the FNR of scale change scenario(6.2%) is relatively higher than that of others. It is because the rear appearance features get ambiguous with increasing distance, thus becomes more uncertain for the detector to discriminate.

**Table 1:** Vehicle detection performance.

Dataset	Scenario	NOV	Ours			VDVP [13]		
			TPR	FPR	FNR	TPR	FPR	FNR
Dataset 1	Dark illumination	1846	94.8%	5.2%	2.4%	86.8%	13.2%	2.4%
Dataset 2	Bright illumination	2537	95.2%	4.8%	3.1%	88.1%	11.9%	3.1%
Dataset 3	Scale change	1748	95.3%	4.7%	6.2%	87.9%	12.1%	6.1%
Dataset 4	Heavy traffic	2215	94.7%	5.3%	2.9%	84.4%	15.6%	2.9%
Average		8346	95.0%	5.0%	3.5%	86.8%	13.2%	3.5%

### Performance of AKF based vehicle tracker

**Fig 10:**
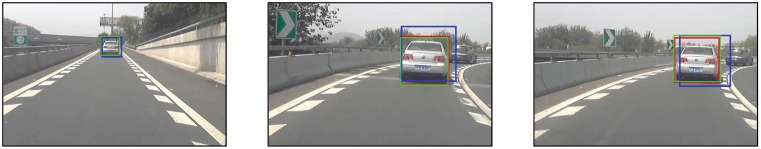
Video sequence of single vehicle with large scale change. Blue-KF, green-GT, red-ours.

To verify the filtering performance improvement introduced by the on-line stochastic modelling of AKF, a typical video sequence of a single vehicle object was filmed. In this sequence, the scale and the location of the target varies due to changing distance and curve lane, see Fig 10. To show the performance improvement, the conventional KF based vehicle tracker used in [12] was used as subject for comparison. The location error and width error are used as comparison metrics, which are respectively defined as

$$location\_error=\sqrt{{\left[\hat{x}\left(k\right)-x_{GT}\left(k\right)\right]}^{2}-{\left[\hat{y}-y_{GT}\left(k\right)\right]}^{2}}$$ (27)

$$width\_error=\hat{w}\left(k\right)-w_{GT}\left(k\right)$$ (28)

where $\left[\hat{x}\left(k\right),\hat{y}\left(k\right),\hat{w}\left(k\right)\right]$ represents the tracked vehicle in the *k*th frame, and [*x*_*GT*_ (*k*), *y*_*GT*_ (*k*), *w*_*GT*_ (*k*)] the ground truth(GT) which has been manually labelled.

**Fig 11:**
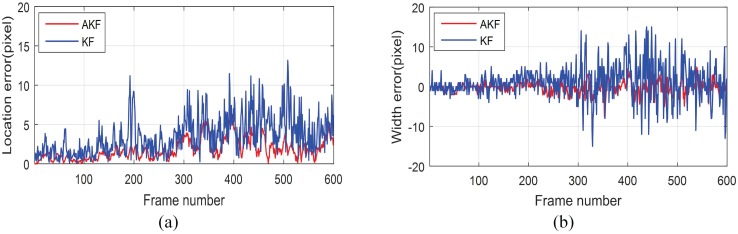
Tracking performance comparison between KF and AKF. (a)Tracking error of location. (b)Tracking error of width.

The tracking errors are as plotted in Fig 11. The maxima, mean and root-mean squares of these errors are shown in Table 2. It could be seen that as the tracking procedure proceeds, the tracking error of KF propagated greatly, while that of the proposed method were efficiently suppressed. It is because the noise property of the measurements changed due to scale and distance variations, and the pre-set process covariance and noise covariance of KF became appropriate. However, with the on-line stochastic modelling, the proposed method could adaptively estimate the covariance of process noise and measurement noise, thus compensate the noise properties changes, and avoid the tracking error propagation.

**Table 2:** Tracking error comparison between AKF and KF.

	AKF based tracker		KF based tracker [12]	
	Location	Width	Location	Width
MaxErr(pixel)	6.3	7.9	13.6	15.1
MeanErr(pixel)	1.8	1.2	3.5	3.1
RMS(pixel)	2.2	1.6	4.3	4.3

### Multi-vehicle tracking performance

To evaluate the multi-vehicle detection and tracking performance systematically, two video clips of typical driving scenarios with dense traffic were tested in this section. These video were captured on the 3-Ring and the 5-Ring road Beijing, which are busy urban express-ways with varying number of vehicles which were appearing, disappearing and reappearing in the field of view(FOV) due to frequent manoeuvres such as cut-in, lane change and overtaking. To better show the improvement of the proposed tracking method, the original vehicle detector with global searching schema was applied for comparison. Considering the detection performance of our detector, the thresholds in the track management were experimentally set as $C_{+}^{T}=5$ and $C_{-}^{T}=4$.

**Fig 12:**
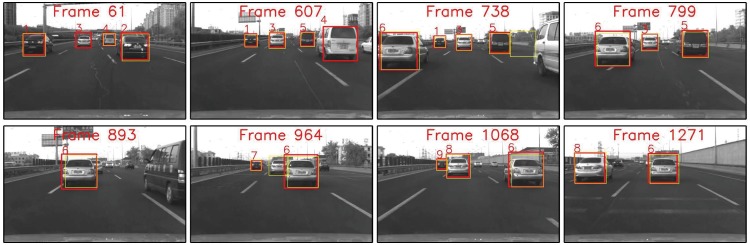
Typical tracking samples of the video captured on 3-Ring road. (Yellow) Original cascade Adaboost detector. (Red) Ours. Note the detection failure and false alarm of original detector in Frame 61 and 738 respectively.

**Fig 13:**
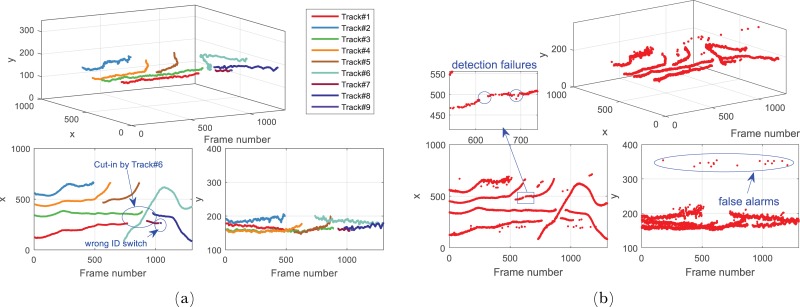
The trajectories of different vehicles in 3-Ring road. (a)The tracking result of our method, and (b)the result of original Cascade Adaboost detector. Note that our method could track each vehicle with correct ID, and efficiently suppress the false positive detections and temporal loss of detection.

The video of 3-Ring road is composed of 1271 frames, and there were 6 different preceding vehicles across three lanes appeared during the tracking procedure. Some of the tracking examples are shown in Fig 12, the tracked trajectories of different vehicle targets are shown in Fig 13, and the full result could be found in the S1 File. It shows that our method could generally track all the relevant vehicle targets continuously and smoothly with correct identities throughout the whole video. Although Track# 1 and Track# 3 were cut by the aggressive lane change manoeuvre of Track# 6, their trajectories were successfully rebuilt by Track#7 and Track#8 after they reappeared. It is worth to note that there was a wrong identity switch from Track# 7 to Track# 9(see Frame 964–1068 in Figs 12 and 13(a)), it is because this target was too small due to the long distance from the ego vehicle, and a short-term detection failures occurs.

**Fig 14:**
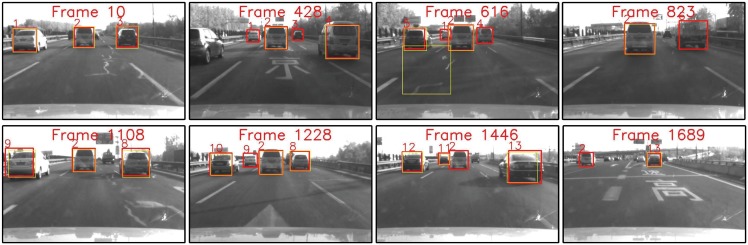
Typical tracking results of the video captured on 5-Ring road. (Yellow) Original cascade Adaboost detector. (Red) Ours. Note that different vehicles are tracked with independent ID numbers throughout the video by our method. Note the detection failures and false alarms of original detector.

**Fig 15:**
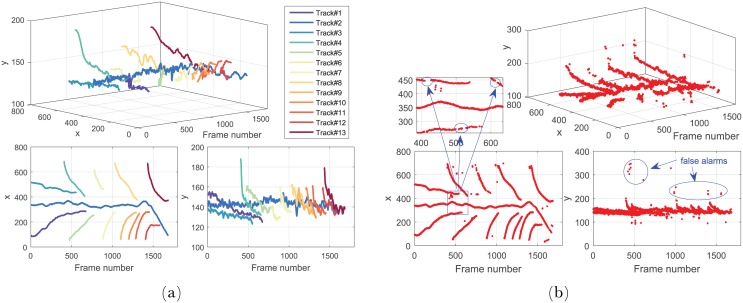
The trajectories of different vehicles. (a)The trajectories of different vehicles produced by our method. (b)The detection result generated by original Cascade Adaboost detector. Our method could track each vehicle with correct ID, and efficiently suppress the false positive detections and temporal loss of detection.

Figs 14 and 15 show the test results of the data captured on 5-Ring road. This video contains 1689 frames, and is featured by frequent overtaking vehicles appearing and disappearing in the FOV(Full result refers to S2 File). There are in total 13 vehicles shown up during the procedure. Sun glares and mottled shadows make the detection more challenging. Comprehensive analysis shows that occasional detection failures and false alarms also occurred in the detection results of the original vehicle detector. And the detection failures are especially severe since vehicles departed(see Frame 428, 616 1227 and 1689 in Fig 14) frequently. However these detection loss and false alarms were also efficiently compensated and eliminated by our method, and more continuous and smooth trajectories with correct identities were generated for each target throughout the sequences. It is worth to note that a few frames of false alarms also occurred in our method(0.2%), it was because of the termination delay caused by tracking mechanism that the object already disappeared due to occlusion, but the track mechanism still held the track for a few frames.

The quantitative results of these two videos are shown in Table 3. Compared with original vehicle detector, our method could achieve a better accuracy with lower processing time. It proves that the proposed data association method could correctly assign measurements with existing tracks in every epoch, the track management method could successfully suppress the sparse false alarms(FPR) and pick up the true detections through temporal context. Furthermore, once a track is created, the track management could efficiently remedy the wrong terminations caused by temporary detection failures. It is worth to mention that most of the remaining detection failure(1.3% FNR) happened during the creation of each track(see Frame 964 in Fig 12 for example) since we only took the registered vehicles for evaluation. Actually this kind of detection failures had already been successfully detected and observed by the track management. Additionally, the ROI based detection with periodical global searching scheme could reduce the computation costs while find the new appearing targets in time, therefore the frame rate is improved remarkably without losing the detection performance compared with the global searching based original detector.

**Table 3:** Multi-vehicle tracking performance.

Scenarios		FPR	FNR	FPS
3-Ring Road	Original Detector	5.2%	3.4%	4.3
	Proposed Method	0.1%	1.4%	23.1
5-Ring Road	Original Detector	3.1%	8.9%	4.7
	Proposed Method	0.2%	1.1%	22.7
Average	Original Detector	4.6%	6.5%	4.5
	Proposed Method	0.2%	1.3%	22.9

### Limitations of the proposed method

**Fig 16:**
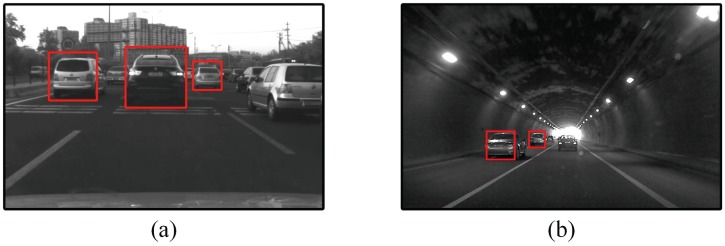
Detection failures. (a)The vehicle in the right lane is not detected due to partial appearance. (b) The vehicle in the ego lane is not detected due to high contrast background.

This section discuss the limitations of the proposed method. As shown in Fig 16(a), the proposed method can only detect the fully appeared or slightly occluded vehicles, when the vehicle targets are highly occluded or partially appeared, it could not guarantee a reliable detection. The proposed detector will also lose the detection when there is high contrast background occurred due to sudden illumination changes, as shown in Fig 16(b). Although the tracking mechanism could mitigate the detection loss to a certain degree, the wrong termination of existing tracks are still usually unavoidable unless the camera’s exposure regains balance very quickly. Future work will focus on improving the detection of these more challenging scenarios. Additionally, the proposed data association and track management method utilized many heuristic fixed thresholds for determining the creation and termination of tracks. Future work will also include analysing the sensitivity and influence of these parameters and finding the adaptive threshold for some important parameters.

## Conclusion

This paper has proposed an improved multi-vehicle tracking method using cascade Adaboost and adaptive Kalman filter with target identity awareness for driver assistant systems. For vehicle detection, we built a cascade Adaboost classifier using Haar-like features, followed by a verification process which could refine the vehicle hypothesis more systematically. In vehicle tracking, the detections of every epoch were assigned by a GNN based association method while considering their local validities, and an adaptive Kalman filter with on-line stochastic modelling was developed for robust state estimation. Additionally, a temporal context based track management was also proposed to suppress spare false detection and avoid wrong termination of existing tracks caused by temporary detection failures. The proposed method was test on various challenging real roads, and the experimental results showed that the vehicle detection performance was greatly improved with lower false alarm rate, lower detection failure rate and higher processing speed. Finally, the limitation of the proposed method and future work were also discussed.

## Supporting information

S1 File The full detection result of 3-Ring Road in video format. (Yellow) Original cascade Adaboost detector, (Red) Ours.
(MP4)

S2 File The full detection result of 5-Ring Road in video format. (Yellow) Original cascade Adaboost detector, (Red) Ours.
(MP4)
